# Supplementary material for: Content-rich biological network constructed by mining PubMed abstracts
Source: BMC Bioinformatics. 2004 Oct 8;5:147. doi: 10.1186/1471-2105-5-147 (PMC528731; doi:10.1186/1471-2105-5-147)
Supplement: Additional File 2 — The original results of the above study (non-essential files are deleted to keep the file size under the limit set by BMC bioinformatics). [file 1471-2105-5-147-S2.bz2 › chilibotAdditionalFile2/dip05/722ID2167176E18062/html/SECE_SECY.html]

 


 **SECE** and **SECY** 
  
Found 94 abstracts in PubMed, retrieved 05.  
 

 What does Google say? 
 PDF only 
| .edu only 

---

**Interactive relationship** (e.g. stimulation, inhibition, etc)

**Neutral relationship**- Although the importance of interactions involving both the cytosolic and transmembrane regions of  **SecY**  and  **SecE**  has been documented, no information has been available for the physical contact sites of these translocase subunits in their cytosolic domains.  Ref: 12809500 Biochemistry, 2003
- Thus, CBD cellulose interactions can be employed as a salt insensitive affinity purification system for the capture of complexes containing the Haloferax volcanii translocation apparatus components  **SecY**  and  **SecE** .  Ref: 12579382 Extremophiles, 2003
- By exploiting the salt insensitive interaction of the cellulose binding domain CBD of the Clostridium thermocellum cellulosome with cellulose, purification of CBD fused versions of  **SecY**  and  **SecE** , components of the translocation apparatus of the halophilic archaeon Haloferax volcanii, was undertaken.  Ref: 12579382 Extremophiles, 2003
- We suggest that the cytosolic and intramembrane interactions bring these two subunits together, forming a functionally crucial SecYE interface involving the  **SecY**  C5 region and the conserved cytosolic segment of  **SecE** .  Ref: 12809500 Biochemistry, 2003
- These cross linkages were abolished by the Gly240 mutation in the  **SecY**  C4 region as well as by prlG alterations in  **SecE**  transmembrane segment 3, known to compromise  **SecY**   **SecE**  interaction.  Ref: 12809500 Biochemistry, 2003

**Non-interactive relationship** (e.g. studied together, co-existance, homology, etc.)

- Overproduction of  **SecE** , but not of  **SecY** , alleviated the dominant negative effects.  Ref: 12655407 Mol Genet Genomics, 2003
- Cysteines introduced into  **SecY**  residue 244 in the fourth cytosolic domain C4 as well as into residues 354 356 and 362 in the C5 domain could be cross linked with natural or engineered residues at positions 79 and 81 in the central part of the cytosolic loop of  **SecE** .  Ref: 12809500 Biochemistry, 2003
- Identification of a  **SecY**   **SecE**  Cytosolic Interface.  Ref: 12809500 Biochemistry, 2003
- Isolation of fusion proteins containing  **SecY**  and  **SecE** , components of the protein translocation complex from the halophilic archaeon Haloferax volcanii.  Ref: 12579382 Extremophiles, 2003
- SecYEG, a complex of integral membrane proteins  **SecY** ,  **SecE** , and SecG in the cytoplasmic membrane, constitutes a pathway for polypeptide movement.  Ref: 12812064 Yi Chuan Xue Bao, 2003
- We now carried out site specific cross linking experiments to identify  **SecY**  and  **SecE**  regions that are physically close.  Ref: 12809500 Biochemistry, 2003
- **SecY** ,  **SecE**  and SecG form a heterotrimer, which functions as a protein translocation channel in Escherichia coli.  Ref: 12655407 Mol Genet Genomics, 2003
- Integral membrane components  **SecY** ,  **SecE** , and SecG of protein translocase form a complex in the Escherichia coli plasma membrane.  Ref: 12809499 Biochemistry, 2003
- Following transformation of Haloferax volcanii cells with CBD  **SecY**  or  **SecE**  encoding plasmids, cellulose based purification led to the capture of stably expressed, membrane bound 68 and 25 kDa proteins, respectively.  Ref: 12579382 Extremophiles, 2003
